# Identification of two novel pathogenic variants of the *NR1H4* gene in intrahepatic cholestasis of pregnancy patients

**DOI:** 10.1186/s12920-022-01240-w

**Published:** 2022-04-18

**Authors:** Hua Lai, Xianxian Liu, Siming Xin, Jiusheng Zheng, Huai Liu, Yu Ouyang, Huoxiu Yang, Yang Zeng, Yang Zou, Xiaoming Zeng

**Affiliations:** 1 Key Laboratory of Women’s Reproductive Health of Jiangxi Province, Jiangxi Provincial Maternal and Child Health Hospital, Nanchang, 330006 Jiangxi China; 2 Department of Obstetrics, Jiangxi Provincial Maternal and Child Health Hospital, Nanchang, 330006 Jiangxi China; 3 Central Lab, Jiangxi Provincial Maternal and Child Health Hospital, Nanchang, 330006 Jiangxi China

**Keywords:** Intrahepatic cholestasis of pregnancy, Nuclear Receptor Subfamily 1 Group H Member 4, Total bile acid, Mutations

## Abstract

**Background:**

Intrahepatic cholestasis of pregnancy (ICP) can cause adverse pregnancy outcomes, such as spontaneous preterm delivery and stillbirth. It is a complex disease influenced by multiple factors, including genetics and the environment. Previous studies have reported that functioning nuclear receptor subfamily 1 group H member 4 (*NR1H4*) plays an essential role in bile acid (BA) homeostasis. However, some novel variants and their pathogenesis have not been fully elucidated. Therefore, this research aimed to investigate the genetic characteristics of the *NR1H4* gene in ICP.

**Methods:**

In this study, we sequenced the entire coding region of *NR1H4* in 197 pregnant women with ICP disease. SIFT and PolyPhen2 were used to predict protein changes. Protein structure modelling and comparisons between NR1H4 reference and modified protein structures were performed by SWISS-MODEL and Chimera 1.14rc, respectively. T-tests were used to analyse the potential significant differences between *NR1H4* mutations and wild types for 29 clinical features. Fisher’s test was conducted to test the significance of differences in mutation frequencies between ICP and the three databases.

**Results:**

We identified four mutations: two novel missense mutations, p.S145F and p.M185L; rs180957965 (A230S); and rs147030757 (N275N). The two novel missense mutations were absent in 1029 controls and three databases, including the 1000 Genomes Project (1000G_ALL), Exome Aggregation Consortium (ExAC) and ChinaMAP. Two web-available tools, SIFT and PolyPhen2, predicted that these mutations are harmful to the function of the protein. Moreover, compared to the wild-type protein structure, the *NR1H4* p.S145F and p.M185L protein structure showed a slight change in the chemical bond in two zinc finger structures. Combined clinical data indicate that the mutation group had higher levels of total bile acid (TBA) than the wild-type group. Therefore, we hypothesized that these two mutations altered the protein structure of *NR1H4*, which impaired the function of *NR1H4* itself and its target gene and caused an increase in TBA.

**Conclusions:**

To our knowledge, this is the first study to identify the novel p.S145F and p.M185L mutations in 197 ICP patients. Our present study provides new insights into the genetic architecture of ICP involving the two novel *NR1H4* mutations.

**Supplementary Information:**

The online version contains supplementary material available at 10.1186/s12920-022-01240-w.

## Background

Intrahepatic cholestasis of pregnancy (ICP) is a pregnancy-specific liver disease characterized by skin pruritus and abnormal liver function, such as elevated liver enzymes and increased serum TBA (≥ 10 μmol/L), that appears in the second and third trimesters of pregnancy [1]. The symptoms and biochemical abnormalities usually rapidly disappear in the early postpartum period [2]. The incidence of ICP disease ranges from 1% to 15.6% depending on geographical location [3–5]. The recurrence rate of ICP in the next pregnancy reaches as high as 40–60% [1]. ICP has been associated with adverse perinatal outcomes, including premature birth and intrauterine death [1, 6, 7]. An elevated level of serum TBA will increase the risk of premature delivery and stillbirth [8, 9]. Therefore, untangling the genetic basis of ICP disease is very important.

Obviously, ICP is a complex disease that depends on multiple factors, including genetic background, metabolites of progesterone, oestrogens, seasons and environmental background [4, 10, 11]. Among them, familial clustering analysis in pedigree studies indicated a genetic predisposition for ICP disease [12–14]. To date, several bile acid homeostasis-related genes, including *NR1H4*, ATP Binding Cassette Subfamily B Member 4 (*ABCB4*), ATP Binding Cassette Subfamily B Member 11 (*ABCB11*) and ATP Binding Cassette Subfamily C Member 2 (*ABCC2*), have been reported. Moreover, multiple previous studies have identified genetic variants of the *NR1H4*, *ABCB4*, *ABCB11* and *ABCC2* genes that contribute to the development of ICP [15–20]. Among them, NR1H4 plays a central role in regulating bile acid metabolism.

NR1H4 is both a key modulator of hepatocyte-protective pathways and a therapeutic target for cholestatic liver disease [21]. *NR1H4* is a BA-activated transporter factor that is responsible for BA homeostasis and acts by binding to DNA response elements through the *NR1H4* DNA binding domain (DBD) in the promoter of target genes (such as *ABCB4*, *ABCB11* and *ABCC2*), thereby activating their transcription [22–24]. Moreover, the C-terminal region of *NR1H4* has a highly conserved ligand binding domain (LBD), which determines the specificity of *NR1H4* ligands. These ligands include farnesoid derivative, BA, unsaturated fat, hepatocyte factor-1 and steroid compound [25, 26]. *NR1H4* has four different isoforms: *α1*, *α2*, *α3* and *α4*. The first two isoforms, which are expressed in the human liver, have a different N-terminus than the other two isoforms [27, 28]. In liver tissue, when raising hepatocyte BA levels, *NR1H4* regulates bile flow by directly inducing gene expression (*ABCB4*, *ABCB11* and *ABCC2*) to stimulate hepatic bile export [29, 30]. Conversely, *NR1H4* represses the expression of bile acid import (*NTCP*) [31] and key enzymes (*CYP7A1* and *CYP8B1*) [32] in the bile acid synthesis pathway through the induction of short heterodimer partner (SHP) [31] in the liver and growth factor 19 (FGF19)/FGF15 [33] in the intestine. In addition, NR1H4^−/−^ transgenic mice exhibited BA pool sizes [34]. Therefore, *NR1H4* maintained a stable TBA level in hepatocytes by regulating TBA synthesis, transport, secretion and metabolism.

Considering that women with ICP exhibited elevated serum BAs and *NR1H4* mutations resulted in altered BA levels, we hypothesized that *NR1H4* mutations might also exist in ICP samples. Here, we recruited a total of 197 Han Chinese women with ICP and analysed the entire coding region of the *NR1H4* gene. A total of 4 mutations, including two novel missense mutations in *NR1H4,* were identified in our ICP samples for the first time.

## Methods

### Samples and features

We recruited 197 patients diagnosed with ICP disease based on clinical symptoms (skin pruritus) and laboratory investigations (fasting TBA ≥ 10 µmol/L, etc.) between 2018 and 2020. Peripheral blood samples from 197 patients with ICP disease were collected from the Department of Obstetrics, Jiangxi Provincial Maternal and Child Health Hospital in Nanchang, China. In addition, we recorded a total of twenty-nine available clinical characteristics, which included age, body mass index (BMI), gestational weeks at diagnosis, gravidity and parity; the level of ion concentration covering K, Na, Cl, Ca, Mg and P; the counts of white blood cells (WBCs), red blood cells (RBCs), platelets (PLTs), and red blood cell distribution width. SD (RDW-SD); the level of serum biochemical indices including TBA, aspartate transaminase (AST), alanine transaminase (ALT), total bilirubin (TBIL), direct bilirubin (DBIL), indirect bilirubin (IDBIL), total cholesterol (CHOL), triglyceride (TG), high-density lipoprotein (HDL), low-density lipoprotein (LDL), uric acid (UA); and the outcomes of pregnant women and newborn babies, including birth weight, bleeding count and Apgar score. The clinical features were determined as described previously [20, 35]. Briefly, the ion concentration and serum biochemical index were examined by an AU5800 automatic biochemical analyser (Beckman Coulter, Inc., USA). Routine blood tests were determined by a Sysmex-xn-2000 automatic blood cell analyser (Sysmex Corporation, Japan).

Summary statistics for all the above clinical features investigated in 197 ICP patients are shown in Table 1. Of these samples, 151 clinical data points were described in our previous study [20, 35]. In addition, 1029 samples without ICP disease were also recruited. The present study followed the tenets of the Helsinki Declaration, and the ethics approval was approved by the Institutional Review Board of Jiangxi Provincial Maternal and Child Health Hospital in China. Each participating woman gave written informed consent (Additional file 1).

**Table 1:** Descriptive statistics of twenty-nine clinical characteristics of 197 ICP patients

Characteristics	N	Mean	SD	Min	Max
Basic information					
Age (years)	197	29.42	5.26	17.00	43.00
Gestational age (days)	192	256.12	23.28	215.00	290.00
BMI (kg/m^2^)	183	25.82	3.92	17.08	38.50
Gravidity (times)	189	2.40	1.55	1.00	8.00
Parity (times)	188	0.63	0.78	0.00	4.00
Serum biochemical index					
K (mmol/L)	187	4.01	0.36	3.20	6.40
Na (mmol/L)	186	137.34	2.21	132.00	143.00
CL (mmol/L)	186	104.08	2.62	97.00	112.00
Ca (mmol/L)	186	2.36	0.17	2.00	2.90
Mg (mmol/L)	186	0.81	0.14	0.60	1.89
P (mmol/L)	186	1.15	0.19	0.70	1.60
WBC (× 10^9^)	196	8.55	2.70	4.11	24.23
RBC (× 10^9^)	196	3.84	0.41	2.96	5.52
PLT (× 10^9^)	196	197.76	58.84	75.00	412.00
RDW-SD (fL)	196	46.09	4.86	36.20	67.30
ALT (U/L)	197	103.14	127.27	3.00	595.00
AST (U/L)	197	87.23	98.98	12.00	509.00
TBA (μmol/L)	197	42.51	38.11	4.20	286.80
TBIL (μmol/L)	195	14.95	7.48	5.70	64.80
DBIL (μmol/L)	195	6.96	6.12	0.90	49.50
IDBIL (μmol/L)	195	8.01	3.48	2.70	26.90
CHOL (mmol/L)	189	6.38	1.67	3.16	13.25
TG (mmol/L)	189	3.61	1.58	1.20	11.10
HDL (mmol/L)	189	1.95	0.50	0.92	4.06
LDL (mmol/L)	189	2.79	1.31	0.04	7.34
UA (μmol/L)	187	326.49	91.76	111.00	701.00
Outcomes of pregnancy women and newborn baby					
Birth weight (kg)	159.00	3.07	0.74	1.23	5.30
Apgar score (1–10)	158.00	9.39	1.24	6.00	10.00
Bleeding count (mL)	156.00	261.89	104.15	90.00	810.00

*ALT* alanine transaminase, *AST* aspartate transaminase, *BMI* body mass index, *CHOL* total cholesterol, *DBIL* direct bilirubin, *HDL* high-density lipoprotein, *IDBIL* indirect bilirubin, *LDL* low-density lipoprotein, *PLT* platelet, *RBC* red blood cell, *RDW-SD* red blood cell distribution width. SD, *TBA* total bile acids, *TBIL* total bilirubin, *TG* triglyceride, *WBC* white blood cell

### Mutation analysis

To excavate the potential mutations of the *NR1H4* gene in 197 samples with ICP disease, we designed a total of nine pairs of primers (Table 2) to sequence the entire coding regions of *NR1H4* through PCR and Sanger sequencing. Briefly, 197 genomic DNA samples were isolated from peripheral blood using an Axy Prep Blood Genomic DNA Mini Prep Kit (Item No. 05119KC3, Axygen Scientific, Inc., Union City, CA, USA). A total of 25 µL PCR system, including 2 µL total 100 ng DNA, 0.5 µL of each forward and reverse primer (2 μM), 12.5 µL mixed comprising Mg^2+^, dNTPs and Taq polymerase (Takara Biotechnology Co., Ltd., Dalian, China), and 9.5 µL ddH2O were mixed in a reaction tube. Touch down procedures were used for PCR amplification as follows: first, DNA was initially denatured at 94 °C for 5 min, followed by 26 cycles of 94 °C for 30 s, (68–0.5) °C for 30 s, and 72 °C for 45 s, after which 19 thermal cycles for 94 °C for 30 s, 55 °C for 30 s, and 72 °C for 45 s, a final extension stage of 72 °C for 10 min, and storage at 4 °C. The obtained PCR products were then examined by 1% agarose gel electrophoresis and sequenced by an ABI 3730 Genetic Analyser (Thermo Fisher Scientific, Inc., Waltham, MA, USA). The potential mutations of the *NR1H4* gene were detected by comparative analysis of 197 samples with ICP disease and 1029 controls without ICP. The mutation site was searched by bidirectional sequencing.

**Table 2:** Primers used for sequencing the coding regions of the *NR1H4* gene

Exon	Amplicon (bp)	Forward primer (5′–3′)	Reverse primer (5′–3′)
1	418	TGAACAGAAACCCACCCT	ATCTCCAACCAAAGTCCC
2	523	ACTCCTAACCATTACGCCAAAC	GCAATTAGTTCAAGGGATTTCA
3	609	TAGTGCTCACTGGCATAG	GTGGTTCATTACCCTTTT
4	553	CTCAAACCTTGGCCTTCC	TTTCTGCTGGCAAACACT
5	415	TCCTGCTGTATTTATGCC	ATCAAGATAGGTGGGAGA
6	487	TGAAGTCTCCCACCTATC	GAACAGACCTGCCTTTCT
7	465	AATGGCAATGATGGTGAT	GTCTTCCTTTGGCTCTTC
8	580	GATTCACTAAATCCCATCT	GCAGAATTATAGGCTACT
9	749	GGCAGAAGCTAGTTGTTA	CTTGAGTGAAACTGGGTA

### Evolutionary conservation analysis

The evolutionary conservative analysis of p.S145Fand p.M185L were performed in 26 representative species, including Chimpanzee, Gibbon, Macaque, Olive baboon, Gelada, Marmoset, Prairie vole, Mouse, Rat, Alpine marmot, Rabbit, Domestic yak, Cow, Goat, Sheep, Sperm whale, Arabian camel, Chacoan peccary, Pig, Dog, Dingo, Cat, Leopard, Horse and Elephant, through the genomic alignments of the Ensembl Genome Browser.

### Protein structural modelling

The protein template of modelling between the reference and modified (p.S145F and p.M185L) mutations of the *NR1H4* gene were conducted using the SWISS-MODEL repository database (http://www.expasy.org/). Then, we compared the protein models simultaneously with the Chimera 1.14rc package.

### Statistical analysis

The *summary* function was used to perform the descriptive statistics on the clinical data of 197 samples with ICP disease. The *t.test* function was conducted to analyse the potential association of 29 clinical data between ICP samples with or without *NR1H4* mutations. The *P* values were two sided, and the results were considered significantly different at *P* < 0.05. The frequency significant difference for *NR1H4* mutations between 197 ICP samples and databases were analysed by *Fisher’s test* function. All the analyses were completed with R software. Logistic regression analysis was performed to assess the clinical parameters (age, gestational age, BMI, gravidity and parity) with the mutations.

## Results

### *NR1H4* mutations

We sequenced 9 exon fragments of the *NR1H4* gene and detected a total of four mutations, including three missense mutations in exons 2, 3 and 4 and one synonymous mutation in exon 5 with 3 samples in 197 ICP patients.

**Fig. 1:**
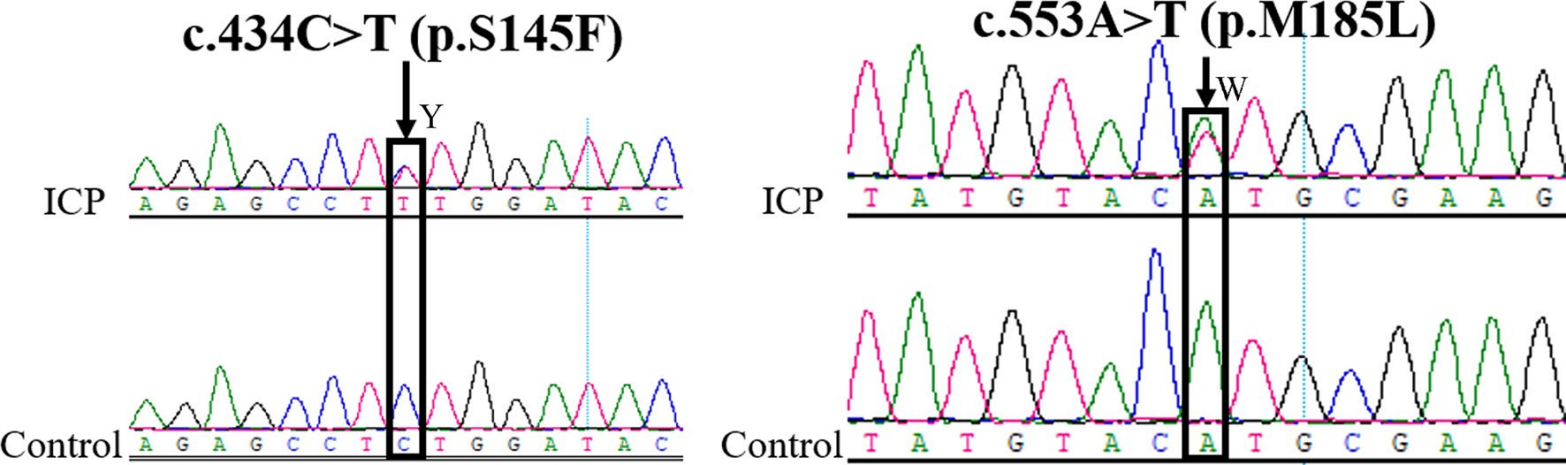
Sequencing electropherograms of two novel mutations (novel-1 and novel-2) in the *NR1H4* gene. The mutation location is marked with an arrow. For novel-1, Y represents C or T in the same sequence. For novel-2, W represents A or T in the same sequence. Novel-1 mutation from C to T and Novel-2 from A to T occurred at the 434^rd^ and 553^rd^ bases in the CDS region of the *NR1H4* gene, respectively. The corresponding amino acids changed from serine (S) to L-phenylalanine (F) in the 145th location and methionine (M) to leucine (L) in the 185th location

Two out of three missense mutations were novel (novel-1, novel-2) (Fig. 1, Additional file 1, Table 3) and were identified in a 40- and 21-year-old ICP individual, respectively. Using the web-available tools SIFT and PolyPhen2, the influence of the two novel mutations on protein function was predicted to be damaging. Furthermore, these two mutations were absent from 1029 controls without the ICP, 1000G_ALL (http://www.internationalgenome.org/), and ExAC (http://exac.broadinstitute.org/) databases. There was a significant difference (*P* = 0.018) in the frequency for two novel mutations between 197 ICP samples and the ChinaMAP (http://www.mbiobank.com/) database.

**Table 3:** Screening for mutations in the *NR1H4* gene in 197 pregnant women with ICP disease

Exon	Patient	SNP	Chr	Position	Alleles	Protein change	SIFT	PolyPhen2	MAF in controls	1000G_ALL	ExAC	ChinaMAP	*P*^1^	*P*^2^	*P*^3^
Exon2	ICP127	Novel-1	12	100904880	C/T	Ser145Phe	0 (D)	0.999 (D)	0/(1029*2)	Not present	Not present	0	–	–	0.018
Exon3	ICP53	Novel-2	12	100926313	A/T	Met185Leu	0.005 (D)	0.981 (D)	0/(1029*2)	Not present	Not present	0	–	–	0.018
Exon4	ICP12	rs180957965	12	100928727	G/T	Ala230Ser	0.815 (T)	0.015 (T)	0/(1029*2)	0.00080	0.00018	0.0029	0.17	0.036	0.44
Exon5	ICP1,69,107	rs147030757	12	100930352	C/T	Asn275Asn	–	–	0/(1029*2)	0.001	0.00022	0.0057	0.0027	1.63e−05	0.11

Significant differences were underlined

*P*^1^ the significance of differences in frequencies between 197 ICP patients and 1000G_ALL, *P*^2^ the significance of differences in frequencies between 197 ICP patients and ExAC, *P*^3^ the significance of differences in frequencies between 197 ICP patients and ChinaMAP

The other missense mutation rs180957965 (p.Ala230Ser) was identified in a 30-year-old sample (ICP12), and the synonymous mutation rs147030757 (p.Asn275Asn) were identified in three ICP patients (ICP1, ICP69 and ICP107). These mutations were all absent in the controls and had a low frequency of databases, ranging from 0.00018 to 0.0057. There was a significant difference in the frequency of the missense mutation rs180957965 (*P* = 0.036) and the synonymous mutation rs147030757 (*P* = 1.63e−05) between 197 ICP patients and the ExAC database. In addition, rs147030757 showed a significant frequency difference between the ICP population and 1000G_ALL (*P* = 0.001).

### Clinical features of ICP patients with *NR1H4* mutations

The clinical and biochemical features of the six ICPs with 4 mutations are presented in Table 4. Serum bile acids were increased in all six patients with *NR1H4* mutations. The serum TBA levels of the patients identified with novel-1 and novel-2 were 46.4 and 113.2 μmol/L, respectively (Table 4). The patient with novel-1 had one child after experiencing six previous pregnancies. The TBA level of the patient ICP12 with the missense mutation rs180957965 was 12 μmol/L, and ICP1, ICP69 and ICP107 patients with a synonymous mutation rs147030757 were had TBA levels of 18.9, 27.5 and 46.4 μmol/L, respectively. Furthermore, the concentrations of CHOL and TG for the six patients with *NR1H4* mutations were higher than the reference values (CHOL: 0–5.2 mmol/L; TG: 0.34–1.69 mmol/L).

**Table 4:** Clinical and biochemical data in the individuals with four mutations covering six patients in the *NR1H4* gene

Characteristics^1^	Novel-1 (ICP127)	Novel-2 (ICP53)	rs180957965 (ICP12)	rs147030757 (ICP1)	rs147030757 (ICP69)	rs147030757 (ICP107)
Basic information^1^						
Age (years)	40	21	30	26	27	27
Gestational age (weeks)	38 + 5	30 + 1	39 + 6	40 + 3	37 + 6	28
BMI (kg/m^2^)	28.2	22	20.4	25.4	24.6	22.2
Gravidity (times)	6	1	2	1	1	5
Parity (times)	1	0	0	0	0	4
Serum biochemical index						
K (3.5–5.1, mmol/L)	4.1	4.3	4.2	4	4.2	3.7
Na (135–145, mmol/L)	137	142	143	135	140	140
CL (96–108, mmol/L)	105	109	104	102	102	108
Ca (2.1–2.9, mmol/L)	2.2	2.2	2.14	2.49	2.34	2.4
Mg (0.6–1.1, mmol/L)	0.8	0.91	0.82	0.74	0.86	0.7
P (0.85–1.51, mmol/L)	1	0.97	1.13	1.37	1.24	0.9
WBC (3.69–9.16, × 10^9^/L)	6.45	7.6	17.59	7.61	5.81	5.49
RBC (3.68–5.13, × 10^12^/L)	3.8	3.31	3.99	3.86	3.8	3.28
PLT (101–320, × 10^9^/L)	164	196	294	144	188	238
RDW-SD (37–54, fL)	51.6	50.4	44.8	57	41.5	43.9
ALT (0–35, U/L)	198	44	76	12	40	7
AST (0–35, U/L)	196	53	51	22	40	15
TBA (0–10, μmol/L)	46.4	113.2	12	18.9	27.5	46.4
TBIL (3.4–20.5, μmol/L)	31.5	10.1	14	13.6	11.9	9.1
DBIL (0–5, μmol/L)	22.4	7.2	6	5	2.5	4.5
IDBIL (0–14, μmol/L)	9.1	2.9	8	8.6	9.4	4.6
CHOL (0–5.2, mmol/L)	5.79	5.52	5.73	6.21	7.44	6.07
TG (0.34–1.69, mmol/L)	3.97	2.47	3.13	2.22	4.89	3.17
HDL (0.9–2, mmol/L)	1.59	1.83	1.6	2.32	2.29	2.27
LDL (0–3.74, mmol/L)	2.4	2.57	2.71	2.88	2.93	2.36
UA (155–357, μmol/L)	339	257	348	282	411	131
Outcomes of pregnancy women and newborn baby						
Birth weight (kg)	3.8	–	3	3.55	3.85	–
Apgar score (1–10)	9	–	8	10	9	–
Bleeding count (mL)	400	–	300	250	350	–

^1^Abbreviations refer to the footnotes in Table 1

### Evolutionary conservative analysis and protein structural modelling

**Fig. 2:**
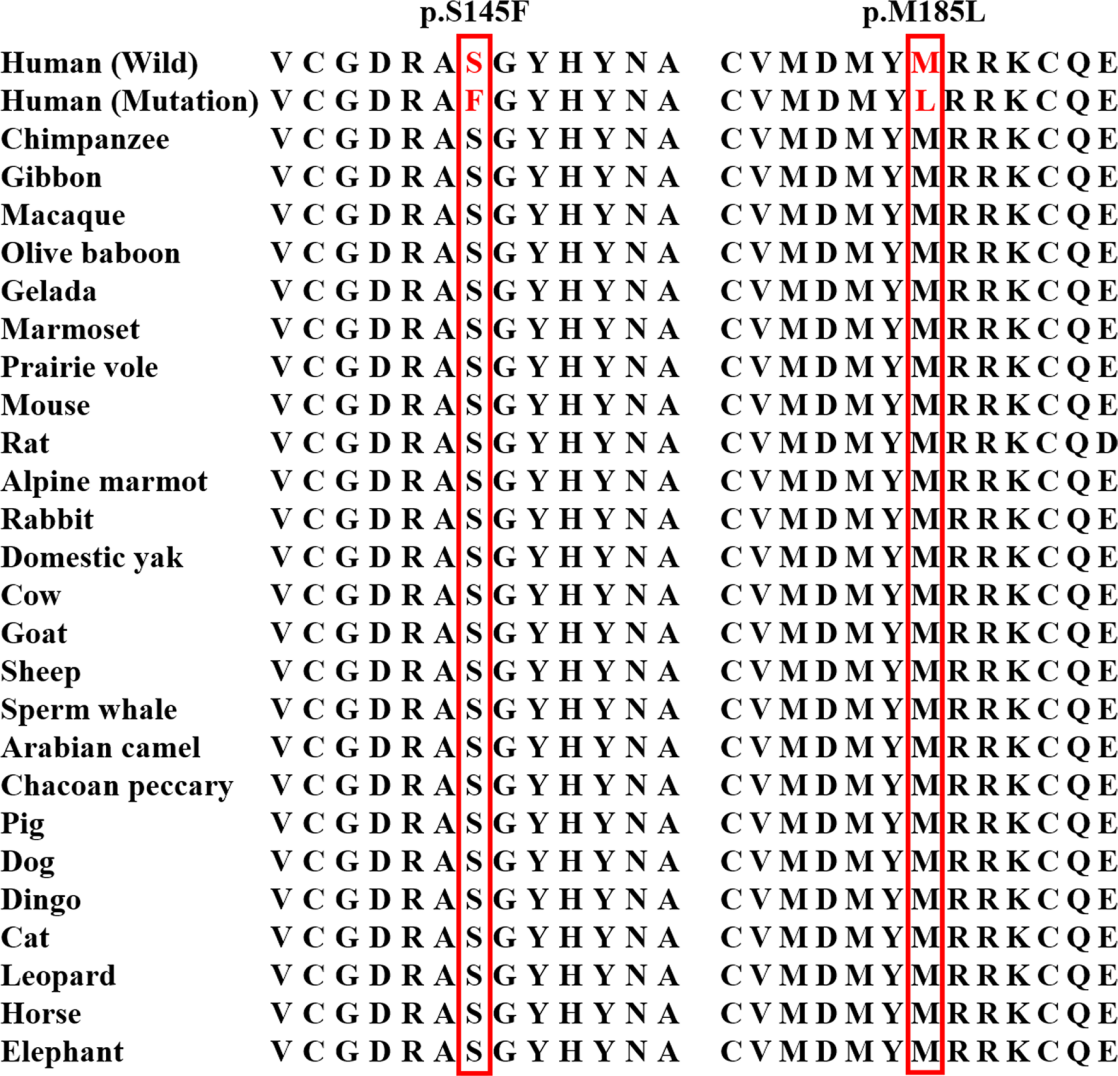
Evolutionary conservation analysis of the *NR1H4* p.S145F and p.M185L mutation among 26 vertebrates, ranging from chimpanzee to elephant. The amino acids serine (S) and methionine (M) in the red horizontal line were highly conserved

Evolutionary conservation analysis showed that these two novel mutations (p.S145F and p.M185L) were highly conserved among the 26 species, ranging from human to elephant (Fig. 2).

**Fig. 3:**
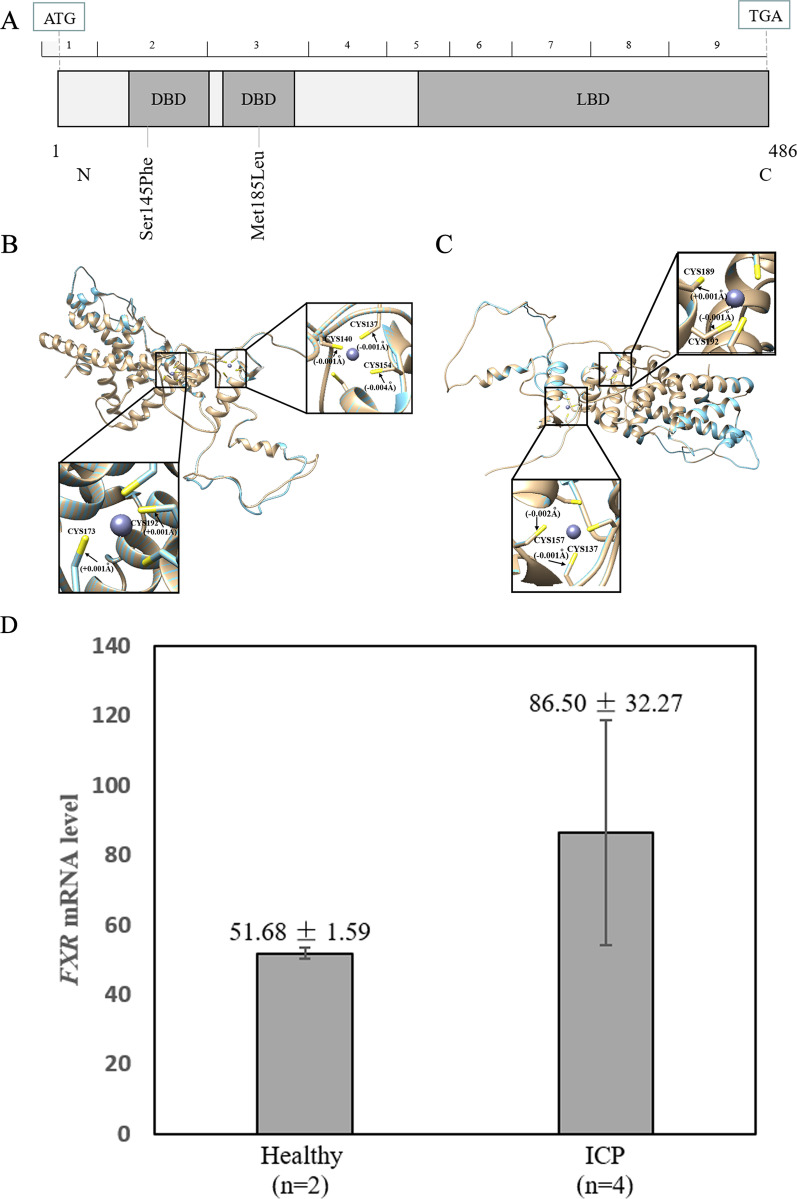
The genetic features of *NR1H4*. **A** The distribution of the *NR1H4* variants. *NR1H4* is a 486-amino acid protein containing two DBD regions and one LBD region. Schematic representation of *NR1H4* NM_001206993.1 cDNA and protein showing the locations of two novel possible pathogenic variants p.S145F and p.M185L detected in two out of 197 patients with ICP disease. Effects of *NR1H4* p.S145F. **B** and p.M185L variants. **C** on the protein structure. The three-dimensional models of reference and modified (p.S145F and p.M185L) NR1H4 showed gold and blue rounded structures, respectively. The enlarged portion showed that the two DBD regions have small changes in the chemical bond lengths. DBD: DNA-binding domain; LBD: ligand-binding domain. **D** Comparison of the expression level of the *NR1H4* gene between two healthy pregnant women and 4 patients with ICP. The expression level of NR1H4 was higher in the ICP group than in the healthy group. The difference did not reach the significance level (*P* = 0.22)

To further investigate the possible effects of the p.S145F and p.M185L variants on protein structure, the reference and the modified protein structure of *NR1H4* gene were compared using UCSF Chimera 1.14rc. These two variants were located in the DNA binding region of the *NR1H4* gene (Fig. 3A). For the variant p.S145F, compared with the reference 3D model of protein structure, the mutation has a slight change in the chemical bond in the two zinc finger structures rich in Cys amino acids at positions 137, 140, 154, 173 and 192 (Fig. 3B). Similarly, for another novel missense mutation, p.M185L, there is a change in the chemical bond at positions 137, 157, 189 and 192 (Fig. 3C).

To further explore the genetic basis of *NR1H4*, we analysed the mRNA expression level of the *NR1H4* gene in placental tissue between two healthy pregnant women and four patients with ICP using NCBI GEO databases (GEO accession: GSE46157) from the Du Q et al. report [36]. The results showed that the expression of *NR1H4* was upregulated in the ICP group (Fig. 3D), even though the difference was not significant (*P* = 0.22).

### Correlation analysis

The potential correlation of *NR1H4* four mutations and 29 available clinical and laboratory data are presented in Table 5. The results showed that the mutation group had higher TBA levels, TBIL levels, and bleeding amounts and a lower Apgar score. In addition, it was found that only the level of Na ions was significantly (*P* = 0.014) higher in the mutation group (139.50 mmol/L) than in the wild-type group (137.27 mmol/L). The associations between the clinical parameters (age: odds ratio (OR) = 0.965; 95% confidence intervals (CI): 0.823–1.132; gestational age (OR = 1.001; 95% CI: 0.982–1.019); BMI (OR = 0.806, 95% CI: 0.605–1.074); gravidity (OR = 1.143, 95% CI: 0.720–1.814); parity (OR = 1.398, 95% CI: 0.572–3,416) and the mutations were shown by logistic regression analysis.

**Table 5:** The potential correlation of *NR1H4* mutations with clinical and laboratory data in 197 ICP patients

Characteristics^1^	Wild type	Mutation	*P* value
Basic information			
Age (years)	29.45 ± 5.24 (n = 191^2^)	28.50 ± 6.35 (n = 6^3^)	0.66
Gestational age (days)	256.29 ± 22.81 (n = 186)	250.83 ± 37.49 (n = 6)	0.57
BMI (kg/m^2^)	25.89 ± 3.94 (n = 177)	23.80 ± 2.83 (n = 6)	0.14
Gravidity (times)	2.39 ± 1.53 (n = 183)	2.67 ± 2.25 (n = 6)	0.67
Parity (times)	0.63 ± 0.75 (n = 182)	0.83 ± 1.60 (n = 6)	0.52
Serum biochemical index			
K (mmol/L)	4.01 ± 0.36 (n = 181)	4.08 ± 0.21 (n = 6)	0.63
Na (mmol/L)	137.27 ± 2.15 (n = 180)	139.50 ± 3.02 (n = 6)	0.014*
CL (mmol/L)	104.04 ± 2.61 (n = 180)	105.00 ± 2.97 (n = 6)	0.38
Ca (mmol/L)	2.36 ± 0.17 (n = 180)	2.30 ± 0.14 (n = 6)	0.37
Mg (mmol/L)	0.81 ± 0.14 (n = 180)	0.81 ± 0.08 (n = 6)	0.87
P (mmol/L)	1.15 ± 0.19 (n = 180)	1.10 ± 0.18 (n = 6)	0.55
WBC (× 10^9^)	8.56 ± 2.64 (n = 190)	8.43 ± 4.58 (n = 6)	0.91
RBC (× 10^12^)	3.84 ± 0.42 (n = 190)	3.67 ± 0.30 (n = 6)	0.32
PLT (× 10^9^)	197.56 ± 59.10 (n = 190)	204.00 ± 54.36 (n = 6)	0.79
RDW-SD (fL)	46.03 ± 4.83 (n = 190)	48.20 ± 5.81 (n = 6)	0.28
ALT (U/L)	104.40 ± 128.54 (n = 191)	62.83 ± 70.74 (n = 6)	0.43
AST (U/L)	88.00 ± 99.84 (n = 191)	62.83 ± 67.00 (n = 6)	0.54
TBA (μmol/L)	42.46 ± 38.25 (n = 191)	44.07 ± 36.68 (n = 6)	0.92
TBIL (μmol/L)	14.95 ± 7.48 (n = 189)	15.03 ± 8.29 (n = 6)	0.98
DBIL (μmol/L)	6.93 ± 6.10 (n = 189)	7.93 ± 7.26 (n = 6)	0.69
IDBIL (μmol/L)	8.04 ± 3.51 (n = 189)	7.10 ± 2.69 (n = 6)	0.52
CHOL (mmol/L)	6.39 ± 1.69 (n = 183)	6.13 ± 0.69 (n = 6)	0.69
TG (mmol/L)	3.62 ± 1.59 (n = 183)	3.31 ± 0.99 (n = 6)	0.63
HDL (mmol/L)	1.94 ± 0.50 (n = 183)	1.98 ± 0.35 (n = 6)	0.84
LDL (mmol/L)	2.80 ± 1.33 (n = 183)	2.64 ± 0.24 (n = 6)	0.77
UA (μmol/L)	327.54 ± 91.69 (n = 181)	294.67 ± 96.65 (n = 6)	0.39
Outcomes of pregnancy women and newborn baby			
Birth weight (kg)	3.06 ± 0.75 (n = 155)	3.55 ± 0.39 (n = 4)	0.09
Apgar score (1–10)	9.40 ± 1.25 (n = 154)	9.00 ± 0.82 (n = 4)	0.24
Bleeding count (mL)	260.82 ± 102.76 (n = 153)	316.67 ± 170.17 (n = 3)	0.32

^1^Abbreviations refer to the footnotes in Table 1

^2^The total number of patients for wild type group

^3^The total number of patients for mutation group

^4^*P < 0.05, the level of Na ion was significantly difference between wild-type group and mutation group

## Discussion

*NR1H4* is required for the basal maintenance of enterohepatic circulation and is responsible for bile acid homeostasis. Milona et al. reported that increased hepatic bile acid concentrations during pregnancy in mice are associated with reduced *NR1H4* function [24], which is consistent with the results of Castano et al. [37]. Castano et al. also demonstrated that impaired *NR1H4* function during pregnancy may be associated with elevated levels of serum bile acids [37]. Our results also found that the expression level of NR1H4 was higher in the ICP group than in the normal group using GEO data. Furthermore, previous studies have demonstrated that functional variants in *NR1H4* are associated with ICP disease/progressive familial intrahepatic cholestasis [16, 21]. In this study, we also detected four mutations, including three missense mutations, S145F, M185L, and rs180957965, and one synonymous mutation, rs147030757. Saskia et al. identified the missense variant M173T in *NR1H4* and conducted cell function analysis [16]. They found that the M173T variant located in the DBD region caused lower transcription levels of bile acid transport-related genes, including *ABCB11* and *IBABP*. In the present study, the two novel mutations S145F and M185L were also located in the first and second zinc finger of the DBD of *NR1H4*. The mutant has a slight change in the chemical bond of the structure for the *NR1H4* gene compared to the wild-type (Fig. 3B, C). Therefore, we speculated that *NR1H4* mutations result in changes in NR1H4 function (Fig. 3D) and the expression level of its target genes, thus increasing the level of bile acids in vivo. The exact mechanism of action remains elusive and requires further experimental study.

To date, an increasing number of researchers have found rare (MAF < 0.01) and low-frequency (0.05 ≤ MAF ≤ 0.01) variants associated with human pregnancy diseases, such as spontaneous preterm birth, cardiomyopathy and preeclampsia, by whole-exome sequencing [38, 39]. Consistent with this, in our study, frequency analysis of all four mutations in *NR1H4* in 197 ICP samples, 1029 controls and 3 website databases covering much larger cohorts suggests that these variants are rare. The allele frequencies of the three missense mutations (MAF = 0.0025) and one synonymous mutation (MAF = 0.007) were lower in this study. According to previous studies, low-frequency and rare variants with large effect sizes contribute to complex traits and diseases [40–42]. Therefore, we hypothesized that the allele frequency and the size effect of mutations have a larger effect on TBA levels. In this study, combining the prediction results with the website available tools SIFT and PolyPhen2 and protein structural modelling, we suspected that the novel mutations contributed more to the development of ICP than the other two. Therefore, it is also likely reasonable that there is no significant difference in TBA levels between wild-type and *NR1H4* mutations even though the mutation group tended to be associated with higher TBA levels when considering the allele frequency and size effect. Except for the ICP caused by the *NR1H4* mutations, we speculated that other gene mutations (such as *ANO8*, ATP-binding cassette transporter family, bile acid receptors) [20, 35, 43], epigenetic regulators (microRNAs, DNA methylation and histone modification) [44–46], oestrogen and progesterone sulfate metabolites [10, 47], hypoxia [48] and the immune system [49], among other factors [50], may be responsible for the remaining ICP patients in this study.

Considering that BAs are toxic to the body, the excessive increase in BA levels has been depicted in different pathological contents. Moreover, several previous studies demonstrated that BAs have the ability to promote lipid absorption and biliary cholesterol secretion [16, 51, 52], indicating that BAs are associated with abnormalities in lipids. Saskia et al. reported that six out of 11 pregnant women with ICP having *NR1H4* variants had symptomatic gallstones [16], and the remaining five did not have gallstone symptoms but had a family history of gallstones. The formation of gallstones is likely determined by the relative concentrations of TBA, CHOL and phospholipids in bile. In the present study, according to the clinical characteristics of 6 ICP cases with *NR1H4* mutations, we found that the TBA levels, CHOL levels and TG levels were higher than the reference values. Therefore, we speculated that these ICP cases with *NR1H4* variants have a high risk for gallstones. Bergheim et al. demonstrated that the possible mechanism of gallstones is the decrease in the expression of the *NR1H4* gene [53]. Furthermore, Moschetta et al. prevented cholesterol gallstone disease by NR1H4 agonists in a mouse model, indicating that *NR1H4* could be associated with cholesterol [54]. In addition, *NR1H4* dysfunctions may occur during the progression associated with inflammatory bowel disease, colorectal cancer in the gut [55, 56], fibrosis and hepatocellular carcinoma in the liver [57, 58]. These results suggest that the variants affecting the structure and functions of NR1H4 lead to gut-liver axis diseases, and in the future, NR1H4 will be proposed as an emerging therapeutic target for both cholestatic and multiple metabolic diseases.

Our present study had several advantages. First, to our knowledge, only a few pathogenic mutations of the *NH1R4* gene, such as M173T, R176* and Tyr139_Asn140insLys, have been identified thus far [16, 21]. Our findings broaden our understanding of the mechanism of NR1H4’s action on ICP disease. Second, *NR1H4* mutations have been detected in ICP families [16, 21]. To date, no studies have uncovered genetic mutations in *NR1H4* genes of hepatic disease among pregnant patients from a relatively large nationally representative sample (n = 197) in China and 1029 local healthy pregnant women. Third, the 29 clinical data of 197 ICP patients are relatively complete, which provides data supporting correlation analysis between mutations and clinical data. However, even though our results provided possible pathogenic variants, the causality between the two potential interesting candidate loci and ICP disease needs to be verified by validation functional experiments.

## Conclusions

In summary, we reported two potential damaging mutations (p.S145F and p.M185L) in the *NR1H4* gene in two out of 197 Chinese patients with ICP for the first time. Our findings provide new insights into the genetic architecture of ICP disease and suggest potential candidate variant targets for ICP clinical treatment.

## Supplementary Information

**Additional file 1.** DNA sanger sequencing electropherograms of two novel mutations (p.S145F and p.M185L) in the* NR1H4* gene.

## Data Availability

The datasets used and/or analyzed during the current study are available from the corresponding author on reasonable request.

## Abbreviations

ICP: Intrahepatic cholestasis of pregnancy
NR1H4: Nuclear Receptor Subfamily 1 Group H Member 4
BA: Bile acid
1000G_ALL: 1000 Genomes Project
ExAC: Exome Aggregation Consortium
TBA: Total bile acid
ABCB4: ATP Binding Cassette Subfamily B Member 4
ABCB11: ATP Binding Cassette Subfamily B Member 11
ABCC2: ATP Binding Cassette Subfamily C Member 2
DBD: DNA binding domain
LBD: Ligand binding domain
BMI: Body mass index
WBC: White blood cell
RBC: Red blood cell
PLT: Platelet
RDW-SD: Red blood cell distribution width.SD
AST: Aspartate transaminase
ALT: Alanine transaminase
TBIL: Total bilirubin
DBIL: Direct bilirubin
IDBIL: Indirect bilirubin
CHOL: Total cholesterol
TG: Triglyceride
HDL: High-density lipoprotein
LDL: Low-density lipoprotein
OR: Odds ratio
CI: Confidence intervals
